# Skin surveillance intentions among family members of patients with melanoma

**DOI:** 10.1186/1471-2458-11-866

**Published:** 2011-11-14

**Authors:** Elliot J Coups, Sharon L Manne, Paul B Jacobsen, Michael E Ming, Carolyn J Heckman, Stuart R Lessin

**Affiliations:** 1 The Cancer Institute of New Jersey, 195 Little Albany Street, New Brunswick, NJ 08901, USA; 2 Department of Medicine, UMDNJ-Robert Wood Johnson Medical School, 125 Paterson Street, New Brunswick, NJ 08901, USA; 3 Department of Health Education and Behavioral Science, UMDNJ-School of Public Health, 683 Hoes Lane West, Piscataway, NJ 08854, USA; 4 Department of Health Outcomes and Behavior, Moffitt Cancer Center, 12902 Magnolia Drive, MRC-PSY, Tampa, FL 33612, USA; 5 Department of Dermatology, School of Medicine, University of Pennsylvania, 2 Maloney Building, 3600 Spruce Street, Philadelphia, PA 19104, USA; 6 Cancer Prevention and Control Program, Fox Chase Cancer Center, 333 Cottman Avenue, Philadelphia, PA 19111, USA; 7 Department of Medicine, Fox Chase Cancer Center, 333 Cottman Avenue, Philadelphia, PA 19111, USA

## Abstract

**Background:**

First-degree relatives of individuals diagnosed with melanoma are at increased disease risk. However, many first-degree relatives do not receive a periodic total cutaneous examination from a health care provider or engage in regular skin self-examination. The goal of this study was to identify correlates of total cutaneous examination and skin self-examination intentions among first-degree relatives of melanoma patients, thus providing insight on factors that should be targeted in future intervention research.

**Methods:**

The participants were 545 first-degree relatives of melanoma patients at increased disease risk due to their risk factor profile and lack of skin surveillance behaviors. Participants completed a telephone survey regarding their total cutaneous examination and skin self-examination intentions and potential correlates, including demographics, medical factors, psychological factors, knowledge, and social influence factors.

**Results:**

Intentions to receive a total cutaneous examination were higher among first-degree relatives with more education, those perceiving higher benefits and lower barriers to an examination, and those reporting greater physician and family support. Intentions to receive a skin self-examination were higher among those with higher benefits and lower barriers to self-examination, and higher family support.

**Conclusions:**

Interventions to promote skin surveillance behaviors among first-degree relatives of melanoma patients should highlight the benefits of early detection of melanoma, address barriers to receipt of total cutaneous examination and engagement in skin self-examination, and promote support from physicians and family members.

## Background

Individuals with one or more first-degree relatives (FDRs) with melanoma have at least a 2-fold increased odds of developing the disease [1]. Early detection approaches for melanoma include total cutaneous examination (TCE) performed by a health care provider and regular skin self-examination (SSE). TCE is a cost-effective, safe, and painless procedure that facilitates identification of thinner lesions that can be treated more successfully than thicker ones [2-4]. Regular engagement in SSE also facilitates early identification of melanoma [5] and may reduce disease mortality [6]. Individuals at increased risk for melanoma, such as due to a family history, are recommended to receive a periodic TCE and engage in regular SSE [7,8]. However, few FDRs of melanoma patients routinely engage in skin surveillance practices [9-13].

The current study sought to identify correlates of intentions to engage in TCE and SSE among FDRs of individuals diagnosed with melanoma. Focus was directed on intentions, because the study targeted individuals who lacked engagement in TCE and SSE and thus may benefit most from interventions to promote these practices. Intentions have been found to mediate associations between health beliefs and various behaviors, including sun protection and receipt of TCE [14,15]. The fact that skin surveillance behaviors are subject to a high degree of personal control and have health promoting, as opposed to health damaging, effects increases the likelihood that changing individuals' surveillance intentions will in turn produce changes in behavior [16].

Drawing from prior studies of correlates of TCE and SSE behaviors [10,13,17], the current study utilized a conceptual framework for examining correlates of TCE and SSE intentions based on the Preventive Health Model [18] and the Theory of Planned Behavior [19]. It was hypothesized that the following factors would be significantly associated with higher TCE/SSE intentions: psychological factors--low fear of TCE (for TCE intentions only), higher benefits and lower barriers to TCE/SSE, and higher distress about the family member's melanoma; social influence factors--greater physician and family recommendations and support for TCE/SSE. The novel correlates examined included fear of TCE, distress about the patient's melanoma, family support for TCE and SSE, and family recommendations for SSE. Similar psychological and social influence factors have been previously linked with other skin cancer prevention behaviors [20], but have not been examined in relation to TCE and SSE intentions or practices among individuals at increased risk for melanoma. The current study provides valuable insight on variables that should be targeted in future interventions.

## Methods

### Participants and procedure

The data were drawn from the baseline questionnaire of a randomized clinical trial to promote skin cancer surveillance and prevention among FDRs of melanoma patients (for more information, see elsewhere [15]). Melanoma patients diagnosed from 3 months to approximately 7 years previously were identified from three medical centers and contacted by mail and telephone. Of the 2310 eligible and locatable probands, 1028 (44.5%) gave permission for medical record abstraction and for their FDRs to be contacted by mail and telephone. A full list of eligibility criteria and recruitment information for FDRs is available elsewhere [15,21]. In brief, eligible FDRs lacked a TCE in the past 3 years, had performed a SSE ≤ 3 times in the past year, had one or more melanoma risk factors, and had no personal skin cancer history. A total of 545 FDRs (50.2% of those eligible and locatable) provided informed consent and completed a telephone survey. Ethical approval for this research was obtained from the Institutional Review Board (IRB) at each study site (Fox Chase Cancer Center, Moffitt Cancer Center, University of Pennsylvania).

### Measures

Responses were averaged across the items of all multi-item scales (for additional information, see Table 1).

**Table 1:** Internal reliability, sample items, and response options for multi-item scales

Scale	Number of items	α	Sample item	Response options
Psychological factors				

Fear of TCE	3	.73	I am afraid of having a total skin examination by a doctor or other health professional.	1 = *strongly disagree *to 6 = *strongly agree*

TCE benefits	12	.90	Having a total skin examination is part of good health care.	1 = *strongly disagree *to 6 = *strongly agree*

TCE barriers	7	.68	It would be embarrassing to have a doctor or other health care professional look at my entire body.	1 = *strongly disagree *to 6 = *strongly agree*

SSE benefits	8	.87	Skin self-examination is very important for people with my history of cancer in the family.	1 = *strongly disagree *to 6 = *strongly agree*

SSE barriers	10	.70	I do not feel confident performing skin self-examination.	1 = *strongly disagree *to 6 = *strongly agree*

Melanoma comparative perceived risk	3	.67	How would you rate your chances of developing melanoma compared with other people with a similar family history of melanoma?	1 = *much lower *to 5 = *much higher*

Perceived severity of melanoma	6	.81	How severely would developing melanoma disrupt your personal health and physical comfort?	1 = *not at all disruptive *to 6 = *extremely disruptive*

Physician/Family support				

TCE physician support	3	.83	I think my doctor's support for having a total skin examination regularly is ...	1 = *no support *to 4 = *a lot of support*

TCE family support	2	.70	My family member with melanoma wants me to have a total skin examination by a doctor or other health care professional	1 = *strongly disagree *to 4 = *strongly agree*

SSE family support	2	.75	My family member with melanoma wants me to do a skin self-examination	1 = *strongly disagree *to 4 = *strongly agree*

Outcome variables				

TCE intentions	4	.92	How likely is it that you will ask your doctor (or other health care professional) to do a total skin examination in the next year?	1 = *not at all likely *to 7 = *extremely likely*

SSE intentions	3	.72	How likely are you to begin doing skin self-examination in the next year?	1 = *not at all likely *to 7 = *extremely likely*

*Note: *TCE = total cutaneous examination; SSE = skin self-examination.

#### Demographics

Participants reported their age, sex, race/ethnicity, education level, marital status, and their relationship to the patient.

#### Medical factors

Participants completed questions about their health insurance and visits to a dentist and doctor in the past year. The number of objective melanoma risk factors for each participant was calculated by summing responses across 5 items. For each FDR, the proband's disease stage and time since diagnosis was noted.

#### Psychological factors

Multi-item measures assessed fear of TCE, TCE benefits, TCE barriers, SSE benefits, SSE barriers [13], comparative perceived melanoma risk [22], and perceived melanoma severity [23]. Single items assessed absolute perceived melanoma risk and level of distress about the patient's melanoma.

#### Knowledge

Single items assessed knowledge of recommended screening frequency for TCE and SSE. Participants also completed 15 true/false melanoma knowledge items [13].

#### Social influence factors: physician and family recommendation and support

A series of items assessed physician recommendations for TCE and SSE (3 items each) [13], physician support for TCE (i.e., participants' perception of the extent to which their physician would want them to get a TCE) (3 items), family recommendations for TCE and SSE (1 item each), and family support for TCE and SSE (i.e., participants' perception of the extent to which family members would want them to be screened) (2 items each).

#### Outcome variables: TCE and SSE intentions

Participants completed multi-item measures of TCE and SSE intentions adapted from prior research [15].

### Statistical analyses

A cutoff of *p *< .05 was used to determine statistical significance. Analyses of SSE intentions focused on the 399 individuals who indicated that they had not done a SSE in the past year. Multiple regression analyses (fit under the assumption of a normal distribution) were used to examine correlates of the two continuous outcome variables, TCE intentions and SSE intentions. Since some participants were members of the same family, the regressions were conducted using a generalized estimating equations (GEE) approach (PROC GENMOD in SAS) with the assumption of an exchangeable correlation matrix and examination of type 3 tests of model effects. For each outcome, a regression analysis was conducted separately for each category of correlates (demographics, medical factors, etc.), with all of the variables in the category included as independent variables. Variables that were specific to TCE or SSE were only included in the respective regression analyses. Separately for each outcome, all of the independent variables that were significantly associated with that outcome across the previous regression analyses were included in a final regression model.

## Results

### Descriptive statistics

The mean age of participants was 46.3 years (*SD *= 13.3), 62.4% were female, 99.1% were white, 56.3% completed college, and 68.1% were married. Most of the participants (56.3%) were the offspring of the melanoma patient, 31.7% were siblings, and 11.9% were parents. On a 1-7 scale, the mean intentions for TCE and SSE were 4.9 (*SD *= 1.8) and 5.0 (*SD *= 1.4), respectively. On average, FDRs reported having a moderate amount of distress about the patient's melanoma (*M *= 2.8, *SD *= 1.2, on a 1-5 scale). The most commonly reported barriers to TCE were: not feeling it necessary to have a TCE unless the person noticed an abnormal growth; inconvenience; embarrassment; and the financial cost. The most commonly reported barriers to SSE were: lack of knowledge of what to look for when doing SSE; preferring a doctor or other health professional check for signs of skin cancer; lack of confidence in how to perform SSE; and not being sure what skin cancer would look like.

### Correlates of TCE intentions

The only statistically significant demographic correlate of TCE intentions was education (parameter estimate [*b*] = 0.20, *SE *= 0.08, *p *= .012). With regard to the medical factors, TCE intentions were higher among participants with more objective melanoma risk factors (*b *= 0.17, *SE *= 0.07, *p *= .018) or those for whom the melanoma patient was diagnosed more recently (*b *= -0.01, *SE *= 0.005, *p *= .010) but did not differ according to participants' health insurance status, the number of visits to a doctor or dentist in the past year, or the disease stage of the melanoma patient. Among psychological factors, TCE intentions were positively associated with TCE fear (*b *= 0.15, *SE *= 0.07, *p *= .029) and TCE benefits (*b *= 0.79, *SE *= 0.11, *p *< .001) and inversely associated with TCE barriers (*b *= -0.79, *SE *= 0.10, *p *< .001). Neither of the knowledge variables was significantly associated with TCE intentions (*p*s ≥ .066). For the social influence factors, TCE intentions were positively associated with physician support for TCE (*b *= 0.46, *SE *= 0.09, *p *< .001), family recommendations regarding TCE (*b *= 0.36, *SE *= 0.17, *p *= .036), and family support for TCE (*b *= 0.52, *SE *= 0.11, *p *< .001). In the final regression model (see Table 2), higher TCE intentions were found among individuals with more education, higher TCE benefits, lower TCE barriers, and greater physician and family support for TCE.

**Table 2:** Multiple regression analysis examining correlates of total cutaneous examination intentions and skin self-examination intentions

Variable	TCE intentions			SSE intentions		
	**Parameter estimate^a^**	**95% CI**	***p *value^b^**	**Parameter estimate^a^**	**95% CI**	***p *value^b^**

Education	0.17	0.04, 0.30	.012			

Number of objective melanoma risk factors	0.09	-0.02, 0.20	.116			

Years since proband's diagnosis	-0.08	-0.17, 0.00	.052			

Fear of TCE	0.11	-0.01, 0.24	.071			

TCE benefits	0.67	0.46, 0.87	<.001			

TCE barriers	-0.72	-0.91, -0.54	<.001			

SSE benefits				0.79	0.63, 0.95	<.001

SSE barriers				-0.25	-0.40, -0.09	.003

TCE physician support	0.25	0.10, 0.39	.001			

TCE family recommendation	0.26	-0.03, 0.54	.081			

TCE family support	0.26	0.06, 0.47	.014			

SSE family support				0.27	0.10, 0.44	.005

*Note: *TCE = total cutaneous examination; SSE = skin self-examination. ^a^Parameter estimates are unstandardized regression coefficients. ^b^*p *values are from type 3 tests of model effects.

### Correlates of SSE intentions

None of the demographic (*p*s ≥ .370) or medical factors (*p*s ≥ .086) were significantly associated with SSE intentions. With regard to the psychological factors, SSE intentions were positively associated with SSE benefits (*b *= 0.86, *SE *= 0.09, *p *< .001) and inversely associated with SSE barriers (*b *= -0.25, *SE *= 0.09, *p *= .009). Knowledge variables were not associated with SSE intentions (*p*s ≥ .101). Among social influence factors, SSE intentions were positively associated with family support for SSE (*b *= 0.47, *SE *= 0.09, *p *< .001). As shown in Table 2, SSE benefits, barriers, and family support remained significantly associated with SSE intentions when they were included in a single regression model.

## Discussion

In this study, we examined correlates of TCE and SSE intentions among FDRs of individuals diagnosed with melanoma. Overall, the levels of TCE and SSE intentions were relatively high (approximately 5 on a 1-7 scale). In line with the study hypotheses, TCE intentions were higher among individuals perceiving higher benefits of having TCE, lower barriers to having TCE, greater physician and family support for TCE, and a higher level of education. Consistent with the correlates of TCE intentions and the hypotheses, individuals with greater SSE intentions had higher SSE benefits, lower SSE barriers, and higher family support for SSE. While perceived benefits, barriers, and physician support have been associated with TCE and SSE behaviors in prior research [10,13,17], the results of the current study suggest that family support may be another important determinant of FDRs' willingness to engage in TCE and SSE. Future research is needed to test family-level interventions using relevant conceptual models of social influence and support. Such research should also seek to identify the optimal approaches for promoting discussions between patients and their FDRs about the importance of engaging in TCE and SSE. Among the demographic factors, the only significant finding was a positive association between education and TCE intentions, which is consistent with studies of TCE receipt in the general population [9,12]. FDRs with a lower level of education should be the focus of targeted interventions to promote TCE. Health care providers and family members should be aware that FDRs with multiple melanoma risk factors, high perceived risk of melanoma, or elevated distress about their family member's illness, will not necessarily be more motivated to engage in melanoma early detection practices.

None of the knowledge variables were associated with TCE or SSE intentions. Simply knowing the recommended frequency of TCE or SSE seemingly does not translate into motivation to engage in the behaviors. Knowledge of melanoma was also not associated with TCE or SSE intentions. Thus, while promoting knowledge of melanoma among FDRs remains an important goal, behavioral interventions should primarily address psychosocial facilitators and barriers of skin surveillance intentions and behaviors. The most strongly endorsed barriers to SSE reflected FDRs' lack of confidence and knowledge in performing SSE, which may most appropriately be addressed by educational efforts and physician support. Overcoming barriers to TCE will require highlighting the importance of regular screening (even if the FDR does not notice an abnormal growth) and addressing practical barriers such as the cost and inconvenience of screening.

### Study strengths and limitations

Strengths of the study include the large sample size and good participation rate (for an intervention study using family referrals for recruitment), the focus on individuals at increased risk for melanoma (due to their family history, risk factor profile, and low engagement in TCE and SSE), and the clinical relevance of the findings. There are several study limitations. The measures were assessed at the same time point, which limits the ability to make causal inferences about the observed associations. The single-item measure of distress we utilized does not provide an indication of the clinical significance of distress. Participants' access to a dermatologist was not assessed. Participants were relatively well-educated, and women and older individuals were more likely to participate. It is not known whether patients who gave contact information for their FDRs differed in any way from patients who did not provide that information.

## Conclusions

This study adds to the small body of literature examining correlates of melanoma early detection intentions and behaviors among FDRs of individuals diagnosed with melanoma. In terms of theory implications, the results of the current study suggest that future research should focus more closely on family-level influence and support factors that may be important determinants of TCE and SSE practices. The study findings suggest that efforts to encourage FDRs to receive a TCE should highlight its benefits in terms of detecting melanoma at an early stage, provide strategies to overcome any perceived TCE barriers, and promote support for TCE from physicians and family members. Individuals with a lower level of education may be most in need of interventions to promote TCE. With regard to promoting SSE among FDRs of melanoma patients, interventions should outline its benefits, attempt to mitigate any perceived barriers (such as lack of confidence or knowledge of how to perform SSE), and encourage family support. Identifying interventions that promote melanoma early detection and prevention practices among FDRs of melanoma patients is an important area for future research.

## Competing interests

The authors declare that they have no competing interests.

## Authors' contributions

EJC designed the study, conducted data analyses, and was the primary author of the manuscript. SLM conceived the study, coordinated data collection, assisted in data analyses, and assisted with writing the manuscript. PBJ participated in the design of the study, coordinated data collection, and assisted with writing the manuscript. MEM coordinated data collection and assisted with writing the manuscript. CJH assisted with the data interpretation and with writing the manuscript. SRL participated in the initial design of the study and coordinated data collection. All authors read and approved the final manuscript.

## Pre-publication history

The pre-publication history for this paper can be accessed here:

http://www.biomedcentral.com/1471-2458/11/866/prepub
